# The prevalence and associated factors of intraoperative hypotension following thoracic surgery in resources limited area, 2023: multicentre approach

**DOI:** 10.1097/MS9.0000000000002665

**Published:** 2024-11-05

**Authors:** Yisehak Wolde, Adugna Argawi, Yabtsega Alemayehu, Mitiku Desalegn, Sintayehu Samuel

**Affiliations:** a Department of Anaesthesia, Wachemo University, College of Medicine and Health Science, Hosanna, Ethiopia; b Department of Anaesthesia, Tikur Anbessa Specialized Hospital, Addis Ababa University, Addis Ababa, Ethiopia; c Department of Anaesthesia, Dilla University, Dilla Town, Ethiopia

**Keywords:** hypotension, intraoperative, prevalence, thoracic surgery

## Abstract

**Background:**

Hypotension is an independent predictor of long-term patient morbidity and duration of hospital stay. Multiple factors contribute to the development of intraoperative hypotension. Prevention and treatment of these factors may reduce patients’ hypotension and its associated morbidity and mortality. This study aimed to assess the prevalence and associated factors of intraoperative hypotension in patients undergoing elective thoracic surgery.

**Methods:**

This institution-based cross-sectional study was conducted among 174 adult patients who underwent elective thoracic surgery. A systematic random sampling technique was used, and quantitative data were collected through interviews and data retrieval from charts via a pretested questionnaire. Both bivariable and multivariable logistic regression analyses were performed to evaluate the associations between independent and dependent variables. The level of statistical significance was defined as a *P*-value less than 0.05. The data were entered into Info 7.2.1 and analyzed via SPSS version 26 software, which was used to calculate descriptive statistics, and bivariate and multivariate logistic regression were performed.

**Results:**

In general, information was collected from 174 patients during the study period. The results of the present study revealed that 65 (41%) patients developed intraoperative hypotension (95% CI: 36.43–48%). Intraoperative blood loss was significantly associated with intraoperative hypotension [AOR=9.58, 95% CI (2.57–35.8)] (*P*=0.001).

**Conclusion and Recommendation:**

The findings of this study revealed high rates of intraoperative hypotension episodes, which were 41%, in patients who underwent elective thoracic surgery. Age, ASA class, type of intraoperative blood loss, type of procedure pre-existence comorbidity, and duration of surgery were predictors of intraoperative hypotension in patients who underwent elective thoracic surgery. The anaesthetist’s, surgeon, and PACU staff’s understanding of these factors is very crucial for close follow-up of this group of patients.

## Introduction

Highlights

- Thoracic surgery is an independent risk factor for perioperative complications, especially in patients with common-morbidities before surgery.
- Hypotension is one of the perioperative complications during surgery.
- Evidence currently available suggests that MAP below 60 mmHg sustained for 5 min or more may be related to organ dysfunction and higher mortality.

Thoracic surgery is a field of medicine that involves the diagnosis and surgical treatment of inside the chest^1^. Thoracic surgery is an independent risk factor for perioperative complications, especially in patients with common morbidities before surgery^2,3^. A thorough preoperative evaluation, smoking cessation, selection of the appropriate surgical procedure, and routine preoperative and postoperative physiotherapy can reduce mortality and major morbidity after lung resection^4–6^.

Several risk factors predict intraoperative hypotension. Patient risk, anaesthesia or surgery-related risk, age, history of medication, ASA physical status, and chronic disease are common patient-related factors. Anaesthetics other than drugs are anaesthesia-related factors. Intraoperative blood loss, surgeon expertise, operation type, and the urgency of the procedure may influence the occurrence of intraoperative hypotension. Cardiorespiratory adverse events may also play a role in the development of hypotension^7,8^.

The prevalence of hypotension in thoracic-surgical patients is high, even in developed countries, at 58.3%^9^. Intraoperative blood loss, age, smoking history, preoperative comorbidity, coronary heart disease, anaesthetic agent use, and the ASA status of patients have been identified as predictive factors^10,11^. There are different definitions of intraoperative hypotension during surgery, with incidences and complications varying accordingly^12,13^.

Recent studies have shown that the definition of intraoperative hypotension varies widely in the literature. In general, an SBP <80 mmHg is considered hypotension. An absolute mean arterial pressure threshold of 65 mmHg was used as the incidence of hypotension: 65% for a greater than or equal to 1 min of exposure, 49% for a greater than or equal to 5 min of exposure, and 31% for a greater than or equal to 10 min of exposure^14^.

Hypotension is the main cause of vasovagal syncope, which leads to loss of consciousness as a result of inadequate blood flow to the brain and a mortality rate of 5–10%^15^. Current evidence suggests that a MAP less than 60 mmHg for 5 min or more may be related to organ dysfunction and increased mortality^16^.

Hypotension is a perioperative complication during surgery. The reviewer agreed that massive surgical haemorrhage (22%) was a significant cause of mortality. Another possible risk factor is the preoperative heart rate (<60 beats/min), preoperative hypotension, advanced age, preoperative renin-angiotensin blockade, revised cardiac risk index (>3 points), and type of surgery. However, other studies have revealed the exact mechanisms by which intraoperative hypotension affects long-term survival not known^17^.

Intraoperative hypotension occurs despite frequent or even continuous intraoperative hemodynamic monitoring, and avoiding it is a complex physiologic challenge for professionals. Recent evidence suggests that a MAP less than 60 mmHg sustained for 5 min or more may be related to organ dysfunction and increased mortality. Interestingly, a recent study revealed that individualised intraoperative blood pressure variability intervention reduces systemic inflammatory response syndrome and organ dysfunction after surgery, although its long-term impact on surgical outcomes has not yet been reported.

## Methods and materials

### Study design and setting

This institutional-based cross-sectional study was conducted in three specialized public hospitals from 15 January to 30 April 2023. Three hospitals were chosen for this study because they are the only government hospitals in which thoracic surgery is performed.

#### Population

All adult patients who underwent elective surgery composed the source population, and all eligible adult patients who fulfilled the inclusion criteria during the data collection period composed the study population. During the preoperative period, patients with an ASA status >IV, patients who refused to provide consent and patients with a history of dementia were excluded from the study.

### Sample size determination and sampling technique

The sample size was determined via the single population proportion method for the first objective, which is the prevalence of intraoperative hypotension during elective thoracic surgery. Since no related study was found in Ethiopia or Africa, *P*=0.5 was used for calculation to obtain the maximum sample size, 95% level of significance, 5% margin of error, and 10% margin of error for incomplete data or contingency data. Population correction was used since the source population was <10 000. Situational analysis of thoracic surgeries was performed 3 months before the start of the study, and an average of 90 procedures were performed per month.

The sample size is calculated as follows:


$$n=\frac{{Z_{\frac{\alpha }{2}}}^{2}P\left(1-P\right)}{d^{2}}.$$

*n*=384.

Since the size of the population is less than 10 000, this study uses a correction formula as follows:


$$\mathrm{Corrected sample size}=\frac{n\times N}{n+N}.$$

*N*=The total number of surgical patients who underwent surgery by general anaesthesia was 270.

The sample size was 158, and adding 10% for the nonresponse rate, the final sample was 174.

A systematic random sampling method was used. Potential study subjects were all eligible patients scheduled for elective thoracic surgery, and their names were selected and included in the surgery list. A list of all adult patients scheduled for thoracic surgery was collected from the theatre lists and submitted to the operating theatre a day prior to the scheduled surgical procedures.

After situational analysis was performed for 3 months before the start of the study, an average of 270 elective surgery cases were found to be performed over 3 months in three hospitals. After proportional allocation, 108 study subjects from one hospital, 39 study subjects from another hospital, and 27 cases also from other hospitals were included. K was determined via the formula K=N/n, where *n*=total sample size and *N*=population. Therefore, the sampling interval was two, and the first study participant (random start) was selected via a lottery method from each daily surgical schedule. Each day before elective surgery begins, patients are selected via a systematic random sampling technique from each surgical schedule. Every second case from the daily surgical procedures was included in the study during the study period.

The intention of including them in the study, the aim and benefits of the study, and the data collection procedures were explained. A preliminary assessment of whether the patient fulfilled the inclusion criteria was performed, and those who met the inclusion criteria were included. This procedure was repeated each day of data collection until the required sample size was attained.

### Operational definitions

American Society of Anaesthesiologists (ASA) physical status classification: This classification was developed by the ASA task force, which classifies patients according to their physical status (systemic well-being)^18^.

ASA class I: normal healthy patient except for the surgical complaint. ASA class II: a patient with mild systemic disease without substantive functional limitations. ASA class III: a patient with a severe systemic disease with substantive functional limitations.

ASA class IV: a patient with severe systemic disease that constantly threatens life.

ASA class V: a moribund patient who is not expected to survive without an operation.

Baseline value: is the value taken before induction.

Hypotension was defined as a ≥30% decrease from baseline or an SBP <80 mmHg^19^.

Hypertension: is an increase of ≥30% from the baseline or an SBP >160 mmHg^20^.

Tachycardia: an increase in heart rate, ≥20% from baseline value^21^.

Bradycardia: a decrease in heart rate ≥30% from the baseline value^16^.

### Data collection tool, method, and procedure

Data were collected via a structured pretested questionnaire by data collectors from 15 January to 30 April 2023. The questions included sociodemographic data, clinical patient characteristics, and different surgical and anaesthetic variables.

Sociodemographic and preoperative clinical data were extracted from the patient’s records on the morning of surgery. Intraoperative anaesthetic and surgical data were collected from the intraoperative anaesthesia charts. Data were collected by three trained anaesthetists; this was done on the morning of surgery, during the intraoperative period, and this process continued until the desired number of patients was achieved.

Medication, comorbidity, and medical history data were extracted from patients’ medical records. Immediately after the patient enters the operating room, the data collector records baseline blood pressure and heart rate before induction and records blood pressure and heart rate postinduction.

Airway pressure values were recorded immediately after the airway was secured. The intraoperative blood loss, type of drug used, amount of fluid or blood taken, and urine output were obtained from the anaesthesia record sheet. Airway pressure (PAP and MAP) and PEEP were monitored by integrated airway pressure monitors on the anaesthesia machines. Blood pressure was monitored via an automatic noninvasive blood pressure monitor and an invasive arterial blood pressure monitoring system. A pulse oximeter and electrocardiograph were used to measure the pulse rate.

### Data quality assurance

To ensure the quality of the data, training on the objectives and relevance of the study and brief orientations on the assessment tools were provided for the data collectors. The questionnaires were prepared in English and pretested on 5% of the study population. The results of the pretest were not included in the final analysis. During data collection, each question was revised by the investigator to be complete and appropriate. In the event of missed measurements during the intraoperative period, the electronic data stored by the monitoring equipment were recalled and backtraced, and the data were filled.

### Data analysis

Data were coded, edited, entered, and cleaned in Epi Info version 7.2 before being exported to the Statistical Package for Social Sciences (SPSS) software version 26.0. SPSS basic descriptive statistics, such as the frequency of preoperative and intraoperative factors, sociodemographic variables, and the prevalence of hypotension, were calculated.

Bivariate and multivariate logistic regression analyses were performed to determine the associations between the dependent and independent variables, and odds ratios with 95% CI were used to determine the degree of association between the dependent and independent variables. The prevalence was calculated as the proportion of patients with intraoperative hypotension with a 95% CI. Variables with a *P*-value less than 0.25 in the bivariate logistic regression analysis were considered for multivariable analysis. After checking for multicollinearity, multivariable analysis was performed to adjust for possible confounders and identify significant predictors. The statistical tests were performed at the 5% significance level. The results are presented in text, tables, charts, and graphs.

This work has been reported in line with the strengthening the reporting of cohort, cross-sectional, and case–control studies in surgery (STROCSS) criteria^22^.

## Results

### Sociodemographic characteristics of patients

One hundred and seventy-four patients were included during the study period. All patients’ records were completely recorded. The results are presented for the all-study variables.

The total numbers of male and female respondents were 109 (62.64%) and 65 (37.35%), respectively. The median age of the patients was 41±13 years. With respect to the American Society of Anaesthesiology, most of the patients had ASAI scores of 47 (27%), ASA II scores of 89 (51.2%), ASA III scores of 24 (13.8%), and ASA IV scores of 14 (8%). Concerning comorbidities, 10 (5.5%) patients had a pulmonary infection, 14 (7.7%) patients had a history of hypertension, 9 (5%) had asthma, 14 (7.7%) had a history of malignancy, and 6 (3.3%) had a history of DM.

### Anaesthetic and surgical characteristics of patients

In total,174 patients underwent surgery under general anaesthesia with endotracheal intubation. Intraoperative anaesthesia was maintained with volatile anaesthesia with halothane and isoflurane and with long-acting muscle relaxants. Fifty per cent of patients received both intermittent boluses of fentanyl and epidural anaesthesia for intraoperative pain management. Paravertebral or erector spinae blocks were used in 27 (15.52%) patients. The majority of surgical procedures lasted more than 2 h (125 (71.2%)).

Invasive monitoring with an arterial catheter was used for blood pressure monitoring in 69 (38.1%) patients. The intraoperative median systolic blood pressure of patients was 109±11, and the lowest intraoperative median systolic blood pressure value was 71±9.

The intraoperative median arterial blood pressure was 67±12. The lowest intraoperative median arterial blood pressure was 50±8. The intraoperative median heart rate was 73±18. The lowest intraoperative median heart rate was 50±7. Treatment of intraoperative hypotension is performed according to the attending anaesthetist or anaesthesiologist.

Noradrenaline was used on 29 (16%) patients, and adrenaline was used on 10 (5.5%) patients for the treatment of intraoperative hypotension. The inhalational agent and beta-blocker were used to treat intraoperative hypertension in 15 (8.3%) patients, and atropine was given to 17 (9.4%) patients to treat intraoperative bradycardia, and lidocaine was used to treat tachycardia in 11 (6.1%) patients.

### Blood pressure variability during the preoperative and intraoperative periods

The baseline median systolic blood pressure of patients was 120±6, and the postinduction median systolic blood pressure value of patients was 105±35. Sixty-five (41%) patients developed intraoperative hypotension, which was a ≥30% decrease from baseline, and the lowest intraoperative median systolic blood pressure was 71±9.

**Figure 1:**
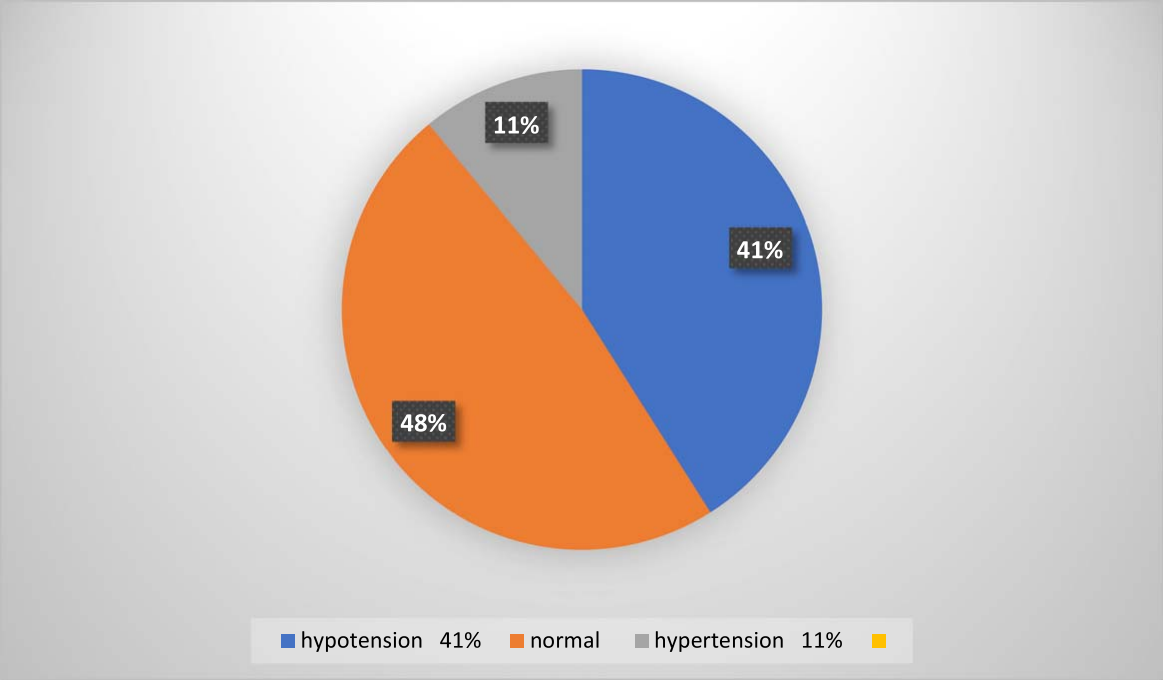
Prevalence of intraoperative hypotension in patients who underwent elective thoracic surgery at the study area.

**Figure 2:**
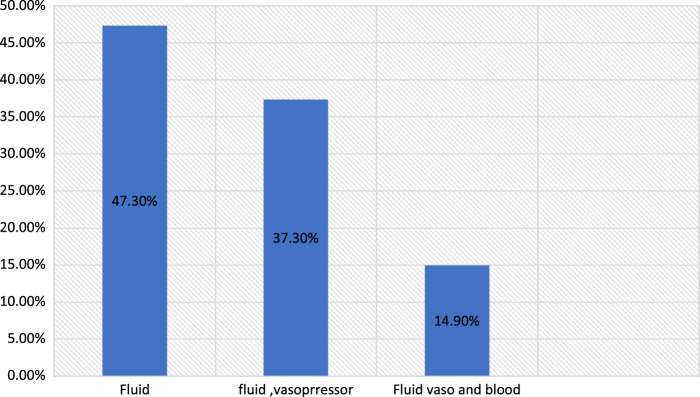
Treatment of intraoperative hypotension in patients who underwent elective thoracic surgery at the study area.

From this postinduction systolic blood pressure value, 16 (8.8%) patients had a decrease of <30% from the baseline value, which was not considered hypotension according to our operational tool. Among the 158 patients, 65 (41%) developed intraoperative hypotension. The lowest intraoperative median arterial blood pressure was 50±7 (Figs 1, 2).

### Associated factors of intraoperative hypotension

Binary logistic regression analysis was conducted to determine the associations between independent variables and intraoperative hypotension. According to bivariate analysis, the presence of intraoperative blood loss with greater than allowable loss, increasing ASA class, and pre-existing comorbidities such as asthma was associated with an increased likelihood of exhibiting intraoperative hypotension.

After excluding variables that did not fit the model with a *P*-value ≥0.25 in the likelihood ratio test, multivariable analysis was performed for selected variables; intraoperative blood loss, type of procedure such as lung pneumonectomy or lobectomy, and hypertension and asthma as coexisting diseases were associated with intraoperative hypotension. There were nine patients with intraoperative blood loss greater than the allowable blood loss (≥10 ml/kg of total estimated blood volume) (AOR=9.584) 95% CI: (2.57–35.8), more likely to develop intraoperative hypotension than patients with less than allowable blood loss (<10 ml/kg of estimated blood volume).

## Discussion

According to this study, there was a greater magnitude of intraoperative hypotension in 41% of patients who underwent elective thoracic surgery. Age, preoperative comorbidity, the use of anaesthetic agents, the ASA status of the patient and intraoperative blood loss were associated with intraoperative hypotension. The higher intraoperative hypotension, may have been related to different factors in our study. Our current study compared with a study done by Sharma *et al*. in India, where the result revealed that 65% of the patients had at least one episode of hypotension^23,24^. This is a higher finding compared to our study, and the possible reason might be study population difference and study area. However, our current study was in line with a study conducted by Korkmaz *et al*., who reported an intraoperative hypotension incidence of 37.6% in adults^25,26^.

The prevalence of the current study findings is similar to those of a cross-sectional study conducted in Iran. After surgery, 36% of patients experienced changes in systolic blood pressure, and 49% experienced diastolic blood pressure changes^27^.

The magnitude of the results of this study is lower than that of a study performed at the University of British Columbia, in which the prevalence of hypotension was 65.8%. The final five risk factors were developed as follows: preoperative heart rate (<60 min), preoperative hypotension (<110/60 mmHg), advanced age, preoperative use of ACE inhibitors or beta blockers, revised cardiac risk index (>3 points), and type of surgery^19^.

Our findings are a lower magnitude of intraoperative hypotension compared with those studies conducted by Zhang, Mu *et al*. at Shanghai Chest Hospital; 58.3% of patients who underwent lung cancer surgery developed intraoperative hypotension. This variation may be attributed to the frequent use of intra-arterial invasive monitoring, which has a better ability to detect intraoperative blood pressure changes. They also used a systolic blood pressure threshold value of less than 100 mmHg for ≥5 min of exposure for hypotension^22,26^. The risk factors for the study conducted above were age, smoking history, preoperative comorbidity, coronary heart disease, the use of anaesthetic agents, the ASA status of the patient, and intraoperative blood loss^10,11^. According to our study results, the risk factors were nearly similar to this finding.

This phenomenon was also observed in a study conducted in Germany on perioperative blood pressure management. For example, in this study, the incidence of intraoperative hypotension varied substantially depending on the selected definition, taking a 20% drop in systolic blood pressure into account, leading to incidences of intraoperative hypotension of 93% for exposures lasting more than or equal to one minute and of 88% for exposures lasting 5 min. Applying an absolute mean arterial pressure threshold of 65 mmHg yielded an incidence of intraoperative hypotension of 65% for a greater than or equal to 1 min exposure, 49% for a greater than or equal to 5 min exposure, and 31% for a greater than or equal to 10 min exposure^14,16^.

Our study also applied an absolute mean arterial pressure threshold of 65 mmHg for greater than or equal to 5 min of exposure and detected the incidence of intraoperative hypotension at 41%, so the findings are similar to study conducted in Germany.

According to our findings, the prevalence of intraoperative hypotension was 41%, and that of hypertension was 11%. This study result was nearly in line with the findings of Gyaninder P. Singh at India Medical College, who studied 298 patients, and Ganjoo *et al*. reported the incidence^25,26^.

In this study, intraoperative blood loss was strongly associated with intraoperative hypotension. The patients who had intraoperative blood loss greater than the allowable blood loss were nine times more likely develop intraoperative hypotension compared with patients whose blood loss less than allowable blood loss. This study result is consistent with that of a study conducted in England involving 676 patients who underwent lung cancer surgery^10,11^.

### Limitations of the study

Preoperative screening for coexisting medical diseases was not performed in some patients because of a lack of standard investigation tools. The only way to identify this group of patients is a variable screening test for each diagnosis. These tests are expensive and are not performed routinely in our setup. Some patients may have been undiagnosed for specific medical diseases. Because these factors may affect the outcome variable, the findings of this study are interpreted with these limitations in mind.

## Conclusion

The findings of this study revealed high rates of intraoperative hypotension episodes, which were 41%, in patients who underwent elective thoracic surgery. Age, ASA class, type of intraoperative blood loss, type of procedure-pre-existence comorbidity, and duration of surgery were predictors of intraoperative hypotension in patients who underwent elective thoracic surgery. The anaesthetist’s, surgeon’s, and PACU staff’s understanding of these factors is crucial for close follow-up of this group of patients. (Tables 1–4).

**Table 1:** Socio-demographic and preoperative clinical characteristics of patients who underwent elective thoracic surgery.

Variables	Category	Frequency
Sex	Male	109 (62.64%)
	Female	65 (37.35.6%)
Age	Group 1, <20	24 (13.3%)
	Group 2, 20–59	114 (63%)
	Group 3, >60	43 (23.75%)
ASA status	Class 1	47 (27%)
	Class 2	89 (51.2%)
	Class 3	24 (13.8%)
	Class 4	14 (8%)
BMI	<25	119 (68.4%)
	25–29	39 (22%)
	>30	16 (9.2%)
Coexisting disease		14 (7.7%)
History of hypertension		
History of asthma		8 (4.4%)
History of DM		6 (3.3%)
History of cancer		14 (7.7%)
History of pulmonary TB		10 (5.512%)

**Table 2:** Intraoperative characteristics of patients who underwent elective thoracic surgery.

Type-procedure	Frequency	Per cent
Esophagectomy	**49**	28.2%
Pneumonectomy	**35**	20.2%
Decortication	45	25.8%
Cystectomy	33	18.9%
Position of patient		
Supine	67	38.5%
Lateral	97	55.74%
Both (lateral and supine)	10	5.74%
Anaesthesia induction and maintenance	Frequency	Per cent
Propofol	127	72.9%
Ketamin and propofol	43	24.7%
Thiopentone	4	2.2%
Halothane	101	58%
Isoflurane	73	41%
Duration of surgery	Group 1, 1–2 h	15 (8.6%)
Duration of surgery	Group 2, 2–4 h	125 (72.9%)
	Group 3, ≥4 h	34 (19.5%)
Intraoperative blood-pressure value (SBP)	1.<80 mmHg	71 (41%)
Intraoperative blood-pressure value (SBP)	2.80–160 mmHg	84 (48.3%)
Mean-arterial pressure (MAP)	3. >160 mmHg 1.<60 mmHg 2.60–120 mmHg 3.>120 mmHg	19 (11%) 71 (41%) 84 (48.3%) 19 (11%)

**Table 3:** Intraoperative characteristics of patients who underwent elective thoracic surgery.

Variables	Median and interquartile range
Fluid given (*n*=174)	2.5±1.5
Urine output (l)	0.9±0.6
Blood loses (l)	1.5±1
Blood given (unit)	2±1
Anaesthesia duration (h)	3±2

**Table 4:** Preoperative and intraoperative factors associated with intraoperative hypotension following elective thoracic surgery.

		Hypotension				
Variables	Category	*Yes*	*No*	COR 95% CI	AOR 95% CI	*P*
BMI	Group 1, <25	53 (42.1%)	73 (57.9%)	1	1	
	Group 2, 25–29	15 (41.5%)	22 (58.5%)	1.01 (0.16–1.47)	0.25 (0.1–3.1)	0.11
	Group 3, >30	4 (36.4%)	7 (63.6%)	0.81 (0.35–11.6)	0.04 (0.01–0.2)	0.17
ASA status	Class I	13 (25%)	35 (75%)	1		
	Class II	40 (47.6%)	41 (52.4%)	2.73 (0.5–5.4)	1.03 (1.196–5.42)	0.97
	Class III	15 (44.11%)	19 (55.88%)	0.86 (0.166–1.173)	1.23 (0.71–3.42)	0.16
	Class IV	6 (54.54%)	5 (45.64%)	1.52 (0.73–3.5)	2.31 (0.45–15.52)	0.38
Type of diagnosis						
Oesophageal cancer	No	60 (48%)	65 (52%)	1	1	
	Yes	14 (39.3%)	34 (60.7%)	0.446 (0.2–2.71)	0.33 (0.095–1.15)	0.05
Lung emphysema	No	61 (37.6%)	101 (62.4%)	1.0	1	
	Yes	13 (68.42%)	6 (31.6%)	3.58 (0.3–5.7)	0.15 (0.03–3.2)	0.02
Lung empyema	No	68 (28%)	92 (71.8%)	1	1	
	Yes	6 (28.6)	15 (71.4%)	0.54 (0.2–38.3)	1.81 (0.44–7.4)	0.41
Mediastinal mass	No	69 (42.8%)	92 (57.1%)	1	1	
	Yes	5 (25%)	15 (75%)	0.44 (0.15–7.03)	0.075 (0.02–1.92)	0.23
Hydatic cyst	No	70 (41.9.%)	97 (58.1%)	1.	1	
	Yes	4 (28.6%)	10 (71.4%)	0.55 (0.1–31.1)	0.6 (0.3–2.912)	0.7
Pre-existing comorbidity						
Hypertension	No	64 (40.7%)	96 (59.3%)	1	1	
	Yes	6 (42.6%)	8 (57.4%)	1.1 (0.33–5.4)	5.48 (0.98–9.09)	0.005
Asthma	No	64 (20%)	104 (80%)	1	1	
	Yes	5 (90%)	1 (10%)	7.68 (0.1–9.91)	0.05 (0.003–0.8)	0.04

Statistically significant *P*<0.05.

AOR, adjusted odds ratio, COR, crude odds ratio.

## Ethical approval

Ethical approval was obtained from the ethical review committee of Addis Abeba University, College of Medicine and Health Sciences. Written informed consent was obtained from each patient, and confidentiality was maintained by making the data collectors aware not to record any identification information found.

## Consent

The patients were informed of the intervention to be performed in their local language. The patient provided written informed consent for publication, including any accompanying photographs. A copy of the written consent is available for inspection by the journal’s Editor-in-Chief upon request.

## Source of funding

Not found.

## Author contribution

Y.W.: contributed to the conception, design, and acquisition of the data; the analysis and interpretation of the data; and the preparation of the manuscript; S.S. and A.A.: was involved in the analysis and interpretation of the data, drafted the manuscript, and critically revised the manuscript for important intellectual content; Y.A. and M.D.: contributed to the design, analysis, critical review, and editing of the manuscript drafts for scientific merit and depth.

## Conflicts of interest disclosure

The authors declare that they have no financial conflicts of interest related to the content of this report.

## Research registration unique identifying number (UIN)

The study was registered in the research registry with a unique identification number of 10334.

## Guarantor

Yisehak Wolde.

## Data availability statement

The data used to support the findings of the study can be obtained from the corresponding author upon reasonable request.

## Provenance and peer review

The paper was not invited and not commissioned. It is an original manuscript submitted to your respected journal.
